# Effectiveness of the Better Operative Software Outcomes Tool for Monitoring Cataract Surgery Outcomes in Inhambane Province, Mozambique: Prospective Pilot Diagnostic Accuracy Study

**DOI:** 10.2196/64500

**Published:** 2025-05-21

**Authors:** Mónica Lecumberri, Dórcia Mandlate, Vasco Cote, Montserrat Martín-Baranera, Carlos L Moser

**Affiliations:** 1 Department of Ophthalmology Consorci Sanitari Integral L'Hospitalet de Llobregat, Barcelona Spain; 2 Eyes of the World Foundation Barcelona Spain; 3 Department of Surgery Universitat Autònoma de Barcelona Barcelona Spain; 4 Coordinator of Research Department Direcção Provincial de Saúde Inhambane Mozambique; 5 Eyes of the World Foundation Inhambane Mozambique; 6 Department of Clinical Epidemiology Consorci Sanitari Integral L´Hospitalet de Llobregat, Barcelona Spain

**Keywords:** cataract extraction, outcome assessment, health care, developing countries, visual acuity, software

## Abstract

**Background:**

Cataract is the world’s leading cause of avoidable blindness. High-quality cataract surgery is a cost-effective procedure to restore vision. In low- and middle-income countries (LMIC), there are high rates of poor outcomes after surgery and inadequate follow-up, which makes it difficult to effectively monitor surgical outcomes. To address this issue, the Better Operative Outcomes Software Tool (BOOST Cataract app) assesses the final outcome on the first postoperative day and records the reasons for poor outcomes at 6 weeks postoperatively, offering specific advice to improve outcomes.

**Objective:**

The aim of this study is to evaluate the real-world utility of the BOOST Cataract app for monitoring cataract surgical outcomes in Inhambane Province (Mozambique) and to analyze the data collected by the app during a surgical campaign conducted therein.

**Methods:**

This prospective study of diagnostic accuracy included patients older than 50 years who underwent manual small-incision cataract surgery. The BOOST Cataract app was used to collect data on visual acuity (VA) on the first postoperative day and to assess surgical outcomes. The sensitivity and specificity (with their 95% CIs) and area under the curve of the BOOST Cataract app (index test) were calculated to identify suboptimal outcomes (moderate and poor) compared with outcomes 6 weeks after surgery (reference standard). The causes of poor outcomes after 6 weeks were recorded and evaluated using the app.

**Results:**

A total of 141 patients who underwent surgery during a cataract campaign in Inhambane Province (Mozambique) between April 2022 and May 2022 were included in the study. The mean age was 70 (SD 10.2) years, and 48.2% (68/141) of the participants were women. Of the 141 patients, 8 (5.7%) did not complete the study. The BOOST Cataract app had a sensitivity of 94.44% and a specificity of 59% for detecting suboptimal outcomes. The area under the curve was 0.825. The campaign outcomes were as follows: good (VA ≥6/18) in 45.9% (61/133) of cases, moderate (VA <6/18 to ≥6/60) in 33.8% (45/133), and poor (VA<6/60) in 20.3% (27/133) of cases. The main cause of a poor outcome was surgical complication, while refractive error was the main cause of a moderate outcome.

**Conclusions:**

The BOOST Cataract app is a valuable tool for assessing suboptimal cataract surgery outcomes without waiting for the completion of postoperative treatment in settings where postoperative follow-up is incomplete. In resource-limited eye care systems, the BOOST Cataract app provides an opportunity to assess outcomes and analyze results to develop specific strategies to improve quality at a local level.

## Introduction

In 2020, cataracts remained the leading cause of avoidable blindness in people aged >50 years, followed by glaucoma and refractive error [1]. Although cataract surgery is a highly cost-effective procedure for restoring vision, only 60% of patients who undergo cataract surgery achieve visual acuity (VA) of 6/18 or better, whereas up to 18% fail to achieve a VA better than 6/60 [2,3]. Surgical outcomes are worse in low-income countries, with an estimated mean effective cataract surgical coverage of 13.9% in the African region [4].

In low- and middle-income countries (LMIC), the low rate of postoperative follow-up makes it difficult to obtain reliable surgical outcome data [5]. The first step toward improving surgical outcomes is to routinely monitor the extent and causes of poor outcomes [6]. In a prospective study conducted at Kikuyu Hospital in Kenya, prospective monitoring improved surgical outcomes [7].

The only known data on cataract surgery outcomes in Inhambane Province came from the Rapid Assessment of Avoidable Blindness (RAAB) study, which was conducted between October 2016 and November 2016. The RAAB study showed that cataract blindness and visual impairment were not controlled in the province, with only 29% of those with blindness (VA <3/60) and 20% of patients with severe visual impairment (VA <6/60) undergoing surgery. Furthermore, only 42.9% of the operated eyes had a good outcome (VA ≥6/18), and 37.5% had a poor outcome (VA <6/60) [8].

Efforts have been made to objectively evaluate surgical outcomes, particularly for residents [9]. Access to the internet in low-resource settings has prompted its use, and it has been suggested that tools such as the Better Operative Software Outcomes Tool (BOOST Cataract app) may facilitate data collection in countries with low postoperative follow-up rates and help improve surgical quality [2].

The BOOST Cataract app was launched in 2018 by a global consortium of leading eye health organizations (the Fred Hollows Foundation, Sightsavers, The International Agency for the Prevention of Blindness, ORBIS International, Aravind Eye Hospitals, and the International Council of Ophthalmology) to address the issues of surgical quality measurement and surgical data recording and to help improve cataract surgical outcomes in low-resource settings.

The BOOST Cataract app is a free and easy-to-use app that can be used for recording and analyzing surgical outcomes the day after surgery. The app does not collect personal patient data but assigns a number to each patient. There are 2 phases in the assessment process. In phase 1, postoperative uncorrected distance VA (BOOST VA) is recorded between 1 day and 3 days after surgery. The BOOST Cataract app estimates the final surgical outcome according to the World Health Organization (WHO) reference criteria, which defines a good outcome as VA ≥6/18 [10] (Table 1). The outcome estimation is based on the direct correlation between VA on postoperative day 1 and the final VA [5,11]. This overcomes the lack of postoperative follow-up, which makes outcome evaluation difficult.

**Table 1:** World Health Organization (WHO) cataract surgery outcome criteria according to postoperative visual acuity.

Snellen PVA^a^				Definition of distance visual impairment (ICD-11^b^)		Standards for postoperative visual acuity according to WHO^c^
**Good**						
	≥6/12		Not impaired		>80%^d^	
	<6/12 to ≥6/18		Mild visual impairment		>80%^d^	
**Suboptimal**						
	**Moderate**					
		<6/18 to ≥6/60	Moderate visual impairment		<15%	
	**Poor**					
		<6/60 to ≥3/60	Severe visual impairment		<5%^e^	
		<3/60	Blindness		<5%^e^	

^a^PVA: presenting visual acuity.

^b^ *ICD-11: International Classification of Diseases, 11th Revision.*

^c^WHO: World Health Organization.

^d^Assigned to only one of the “Not impaired” or “Mild visual impairment” categories, not both at once.

^e^Assigned to only one of the “Severe visual impairment” or “Blindness” categories, not both at once.

Phase 2 consists of analyzing the possible causes of poor outcomes at 6 weeks postoperatively. For this purpose, the presenting visual acuity (PVA) and best-corrected distance visual acuity (BCVA) of 20 patients with poor outcomes at least 6 weeks after surgery are recorded, and the possible etiologies (selection, complication, and refractive error) are assessed. In very low-resource settings where refractive services are unavailable, VA obtained with pinhole (Pin VA) is accepted as an approximation of BCVA. Based on these data, the tool can then identify the main cause of poor outcomes and suggests specific interventions aimed at improvements [12]. The BOOST Cataract app has been used in several countries to monitor the quality of cataract surgery [13]. A study evaluating the app’s effectiveness at predicting the final surgical outcome showed that the ability of the BOOST Cataract app to correctly categorize surgical outcome was dependent upon the presence of corneal edema on postoperative day 1. However, this study was retrospective and not based on an LMIC population [14].

This study aimed to evaluate the utility of the BOOST Cataract app (software version 1.1.2) in a surgical campaign conducted in Inhambane Province and to perform an in-depth analysis of the outcomes and causes of suboptimal results.

## Methods

### Participants and Settings

Consecutive patients presenting with cataracts, who were aged >50 years, and who attended the Provincial Hospital of Inhambane and the District Hospital of Zavala, Mozambique, between April 2022 and May 2022 were included. Patients with cataract were recruited using convenience sampling according to the hospital’s inclusion protocol for cataract surgery: patients aged >50 years with VA ≤6/60 in 1 eye and NC5 or NC6 cataract. The exclusion criteria were the absence of a pupillary light reflex and retinal pathology demonstrated by fundoscopy (NC5 cataract) or ultrasound (NC6 cataract), corneal scars affecting the visual axis, and advanced glaucoma demonstrated by optic nerve examination when it was possible to obtain.

### Ethics Approval

Informed written consent was obtained from all patients who underwent surgery. All data were anonymized. As the study was carried out in the setting of standard clinical practice, the patients did not receive any financial compensation. This study adhered to the tenets of the Declaration of Helsinki and was approved by the Institutional Bioethics Committee for Health of the Republic of Mozambique (CNBS; reference: 443/CNBS/23).

### Preoperative Examinations

All patients underwent a complete ophthalmological examination, including PVA, slit-lamp examination, pupillary reflexes, Goldmann applanation tonometry, and fundus examination, if lens opacity allowed. The power of the implanted intraocular lens (IOL) was calculated using a keratometer (LGM OftalTech) and mode A ultrasound (Scan sw-1000; Suoer). Patients for whom keratometry or biometry could not be performed were assigned to the IOL of the contralateral eye, if possible, or a standard 21 D IOL, if not.

### VA Assessment

PVA was assessed using Tumbling E charts for distance vision, with available correction. After correctly identifying the direction of more than one-half of the optotypes in the upper line, the patient moved to the next line then to the lower lines. The lowest line at which more than one-half of the optotypes were read was recorded as the patient’s VA. The patient moved one-half the distance when the direction of any line could not be identified. If the patient was unable to identify the optotypes at that distance, the assessor’s fingers were placed 3 meters, 2 meters, or 1 meter from the patient. If the patient was unable to see their fingers, a light source was placed to document light perception.

### Surgery and Postoperative Treatment

All surgeries were performed under retrobulbar anesthesia by the same ophthalmologist with extensive experience in manual small-incision cataract surgery (MSICS). MSICS was performed using a superior scleral tunnel. A rigid PMMA biconvex posterior chamber IOL (S3550 Aurolens; Aurolab Inc) was implanted with capsular support. Eyes with zonulolysis or extensive capsular rupture were implanted with an A5520 anterior chamber lens (Aurolab Inc). At the end of surgery, all patients received perioperative prophylaxis with intracameral 0.1 mL cefazolin (2.5 mg/mL) or 0.1 mL vancomycin (0.1 mg/mL) if they were allergic to penicillin [15].

All patients followed the same postoperative treatment protocol at the hospital, consisting of tapering doses of MFLOX-DM (moxifloxacin 0.5% + dexamethasone sodium phosphate 0.1%; Aurolab Inc) for 4 weeks. Eyes with intraocular pressure higher than 25 mm Hg received acetazolamide (250 mg Diamox).

### Assessment the Day After Surgery and Phase 1 of BOOST Cataract App

In phase 1, patients were examined the day after surgery, and VA was measured in the operated eye by an independent observer (BOOST VA). The BOOST VA was entered into the app, and the surgical outcomes were calculated according to the WHO criteria (Table 1). 

Slit-lamp examination was also performed to detect corneal edema. Significant corneal edema was defined as >10 Descemet folds or stromal corneal edema affecting the visual axis.

### Assessment After 6 Weeks and Phase 2 of the BOOST Cataract App

All patients were instructed to return for follow-up 6 weeks after surgery and received a telephone call 2 days before the visit as a reminder to ensure complete follow-up after surgery.

All patients who completed follow-up at 6 weeks had their uncorrected VA (PVA), BCVA, and PinVA (pinhole visual acuity) assessed by an optometrist masked to the BOOST VA. A biomicroscopic examination and autorefraction analysis were performed (LGM OftalTech).

The VAs obtained at 6 weeks were classified according to the WHO criteria (Table 1): without optical correction (PVA outcome) and with pinhole (PinVA outcome).

In phase 2 of the BOOST Cataract app, the cause of poor outcomes (VA <6/60) was identified and recorded as selection, complications, or refractive error. The same was done for patients with a moderate outcome (VA <6/18 to ≥6/60).

### Statistical Analyses

Statistical analyses of the data were performed using SPSS Statistics v21 (IBM Corp). Absolute and relative frequencies were obtained for qualitative variables. For the quantitative variables, depending on whether the normal distribution criteria were met, the mean and SD, median, IQR, and minimum and maximum values were considered.

Regarding the different VAs collected, an equivalent in decimal notation was assigned to the VA that could not be expressed numerically (light perception, counting fingers) [16,17]. The decimal values were converted to a LogMAR (minimum angle-of-resolution) scale for subsequent statistical analysis.

To analyze the ability of the BOOST Cataract app to predict the final surgical outcome, the results obtained on postoperative day 1 using the app (BOOST outcome) were compared with those obtained 6 weeks postoperatively (PVA outcome). For this analysis, only cases with complete follow-up were included. Both variables were grouped into 2 categories (good and moderate/poor results). Using the 6-week results as the reference standard, the sensitivity, specificity, positive predictive value, and negative predictive value of the BOOST Cataract app as a tool for predicting a negative result (moderate or poor) were estimated. All estimates are presented with the corresponding 95% CIs. A receiver operating characteristic curve was generated, and the area under the curve was calculated.

The presence of corneal edema on day 1 was tested as an independent predictor of moderate or poor outcomes at day 1 and at 6 weeks after surgery using a logistic regression model.

## Results

### Characteristics of the Campaign

**Figure 1:**
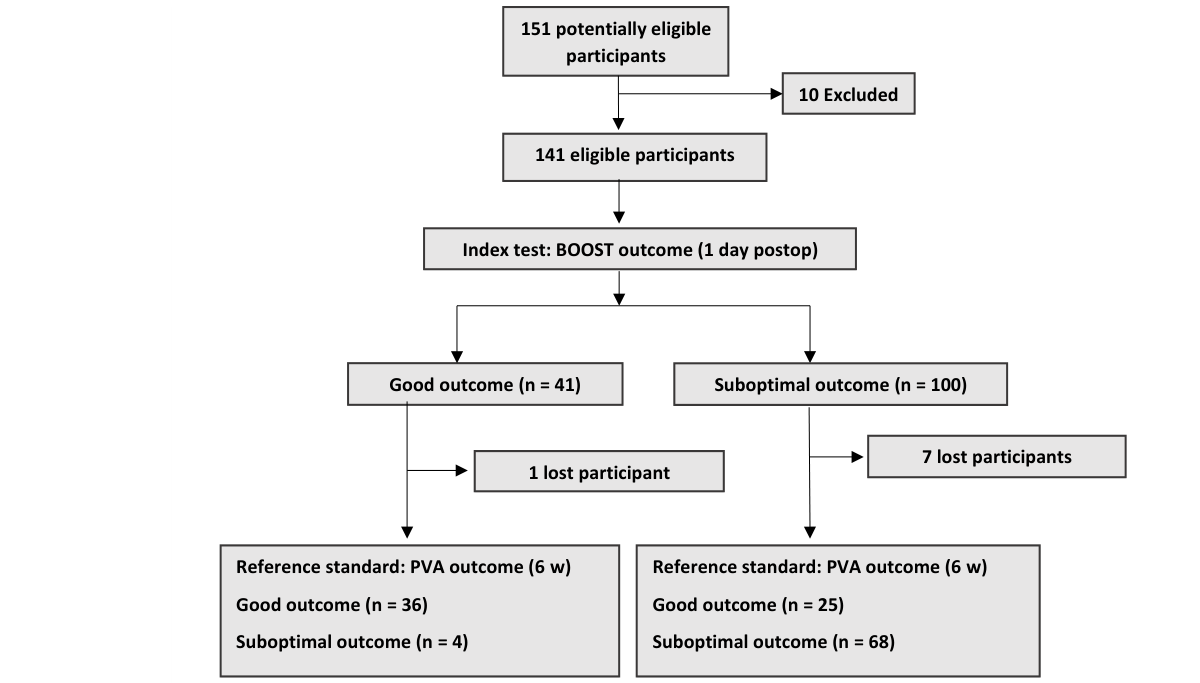
Flow diagram of participants. BOOST: Better Operative Software Outcomes Tool; PVA: presenting visual acuity.

We recruited 151 patients, and 10 were excluded because they did not meet the inclusion criteria for surgery. A study flowchart is presented in Figure 1.

The study included 141 eyes of 141 patients who underwent surgery at the study site: 68 patients (68/141, 48%) were women, and 62 patients (62/141, 44%) were blind (VA <3/60 in the better eye; Table 2). This was the first eye operated on in 108 of 141 (76.6%) patients. In addition, 14 of 141 (9.7%) patients had a history of glaucoma, and 3 of 141 (2.1%) eyes had phacodonesis on biomicroscopic examination. In 123 of the 141 (87.2%) operated eyes, it was not possible to perform a fundus examination because of cataract density. Biometry could not be performed in 22 patients because of corneal irregularities or lack of fixation.

**Table 2:** Preoperative data.

Characteristic			Total sample (n=141)		Women (n=68)		Men (n=73)
Age (years), median (SD)			70 (10.2)		70.28 (9.7)		69.77 (10.6)
**Preoperative Snellen visual acuity, n (%)**							
	<3/60	86 (61)		42 (618)		44 (60.3)	
	≥3/60 to <6/60	52 (36.9)		24 (35.3)		28 (38.4)	
	≥6/60	3 (2.1)		2 (2.9)		1 (1.4)	
**Contralateral eye status, n (%)**							
	Aphakia	1 (0.7)		0 (0)		1 (100)	
	Phakic eye	108 (77.1)		51 (47.2)		57 (52.8)	
	Pseudophakia	31 (22.1)		17 (54.8)		14 (45.2)	
**Visual status, n (%)**							
	Blindness^a^	62 (44.6)		27 (43.5)		35 (56.5)	
	Severe visual impairment^b^	13 (9.4)		8 (61.5)		5 (38.5)	
	Moderate visual impairment^c^	12 (8.6)		8 (66.7)		4 (33.3)	
	Mild visual impairment^d^	32 (23)		18 (56.3)		14 (43.8)	
	Not impaired^e^	20 (14.4)		7 (35)		13 (65)	

^a^<3/60 in the better eye.

^b^≥3/60 to <6/60 in the better eye.

^c^<6/18 to ≥6/60 in the better eye.

^d^<6/12 to ≥6/18.in the better eye.

^e^≥6/12 in the better eye.

### Outcomes of the Campaign: Phase 1 of BOOST Cataract App

Of the 14 intraoperative complications observed, 11 (7.8% of the 141 surgeries) were capsular ruptures. In addition, 10 (7.1%) anterior chamber IOL were implanted, and 1 case of aphakia was reported.

The outcomes of the BOOST Cataract app (BOOST outcomes) are presented in Table 3. Overall, 29% (41/141) of the operated eyes showed good outcomes. Of the 141 eyes, 74 (52.2%) had corneal edema on postoperative day 1. Corneal edema was associated with suboptimal (moderate and poor) BOOST outcomes (ρ<0.001).

The surgical outcome on day 1 (BOOST outcome) was worse than at 6 weeks. Of the 141 patients, 8 (5.7%) did not attend the 6-week postoperative visit. Of these, 6 underwent uneventful surgery. Of the patients who did not complete the follow-up period, 50% (4/8) had poor BOOST outcomes.

The final outcomes (PVA outcome) were good for 45.9% (61/133) of the eyes and remained poor for 20% (27/133) of the eyes. The PinVA outcomes were good for 73% (94/128) of the eyes and poor for 13% (17/128) of the eyes (Table 3).

Eyes that had a suboptimal outcome with the BOOST Cataract app were 21.44 (95% CI 6.35-72.35) times more likely to have a moderate or poor outcome at 6 weeks postoperatively compared with the eyes with a good outcome (ρ<0.001). Corneal edema was associated with the BOOST outcome (ρ<0.001) but not with the final PVA outcome (ρ=0.58).

**Table 3:** Surgical campaign outcomes.

Outcomes				Total sample		Women		Men			
**Postoperative day 1**											
	**BOOST^a^ outcome (n=141), n (%)**										
		Good^b^	41 (29.1)		—^c^		—				
		Suboptimal	100 (70.9)		—		—				
		Suboptimal: moderate^d^	53 (37.6)		—		—				
		Suboptimal: poor^e^	47 (33.3)		—		—				
**6 weeks postoperative**											
	**PVA^f^ outcome (n=133), n (%)**										
		Good	61 (45.9)		30 (46.2)		31 (45.6)				
		Suboptimal	72 (54.1)		35 (53.8)		37 (54.4)				
		Suboptimal: moderate	45 (33.8)		22 (33.8)		23 (33.8)				
		Suboptimal: poor	27 (20.3)		13 (20)		14 (20.6)				
	**PinVA^g^ outcome (n=128), n (%)**										
		Good	94 (73.4)		43 (69.4)		59 (77.3)				
		Suboptimal	34 (26.6)		19 (30.6)		15 (22.7)				
		Suboptimal: moderate	17 (13.3)		11 (17.7)		6 (13.6)				
		Suboptimal: poor	17 (13.3)		8 (12.9)		9 (9.1)				
	Postoperative spherical equivalent (D^h^), median (range)		–1.38 (–9.62 to +1.25)		—		—				
	Postoperative astigmatism (D), median (range)		–3 (–0.5 to –8.75)		—		—				

^a^BOOST: Better Operative Outcomes Software Tool.

^b^Visual acuity (VA) ≥6/18.

^c^Not applicable.

^d^VA: <6/18 to ≥6/60.

^e^VA: <6/60.

^f^PVA: presenting visual acuity.

^g^PinVA: visual acuity with pinhole.

^h^D: diopters.

### Diagnostic Accuracy of the BOOST Cataract App

The sensitivity of the BOOST Cataract app for identifying suboptimal outcomes was 94%. The specificity was 59%. The area under the curve was 0.825 (Table 4).

**Table 4:** Diagnostic accuracy of the Better Operative Outcomes Software Tool (BOOST Cataract app).

Performance measures	Results, % (95% CI)
Sensitivity	94.44 (86.38-98.47)
Specificity	59.02 (45.68-71.45)
Positive predictive value	73.12 (66.69-78.70)
Negative predictive value	90.00 (77.24-95.98)

### Reasons for Suboptimal Outcomes: Phase 2 of the BOOST Cataract App

Surgical complications (capsular rupture and zonulolysis with vitreous loss) were the main causes of poor final outcomes, while refractive error was the main cause of a moderate outcome (Table 5).

**Table 5:** Causes of a suboptimal outcome according to phase 2 of the Better Operative Outcomes Software Tool (BOOST Cataract app).

Outcomes			Results, n (%)
**Poor outcome^a^ (n=27)**			
	Selection	7 (26)	
	Refraction	8 (30)	
	Complication	12 (44)	
**Moderate outcome^b^ (n=45)**			
	Selection	4 (9)	
	Refraction	36 (80)	
	Complication	5 (11)	

^a^Visual acuity (VA) <6/60.

^b^VA <6/18 to ≥6/60.

## Discussion

### Principal Findings

The BOOST Cataract app was able to detect final suboptimal outcomes (VA <6/18) on the day after surgery with a sensitivity of 94%. Due to this sensitivity, it was possible, with reasonable confidence, to identify eyes that were moderately visually impaired or worse at the end of postoperative treatment.

Resource-poor LMIC are often characterized by a lack of equipment, high staff turnover, and frequent surgical campaigns where many surgeries are performed in short periods. In this context, the routine use of the BOOST Cataract app ensures that any decrease in quality is detected early in phase 1.

The specificity of the campaign in this study was only 59%. This low specificity did not provide sufficient certainty estimating the extent of good outcomes. Therefore, the BOOST Cataract app did not determine the center’s local effective cataract surgical coverage. This is because it underestimated good outcomes in phase 1 by not considering transient corneal edema that occurs in the immediate postoperative period and usually resolves without sequelae.

At 6 weeks postoperatively, 45.9% (61/133) of the operated eyes had good outcomes, and just over 20% (27/133, 20.3%) did not achieve vision better than 6/60; with pinhole, 73.4% (94/128) had a good outcome, and 13.3% (17/128) had a poor outcome. These results do not meet the quality standards recommended by the WHO (Table 1).

In the 2016 RAAB study conducted in the province, 42.9% of patients aged >50 years who underwent cataract surgery had good outcomes, and as many as 37.5% had poor outcomes; among patients assessed using PinVA, only 55.4% had good outcomes, and 34.8% had poor outcomes [8].

The 2016 RAAB study collected information on surgeries performed before 2016 when the sutureless extracapsular technique was still being introduced. MSICS is now fully established, and surgical outcomes seem to have improved. However, the RAAB study showed outcomes over time, whereas our hospital-based study only observed outcomes at 6 weeks and may not have captured long-term surgical outcomes and complications. A new RAAB study in the province is required to demonstrate the improvement in surgical outcomes.

In phase 2 of this study, surgical complications were the main cause of poor outcomes (12/27, 44%). In Inhambane Province, as in other provinces of Mozambique, cataract surgery is performed in patients with a preoperative VA ≤6/60 [18]. It is common for cataracts to present at very advanced stages with a higher rate of complications (zonulolysis and capsular rupture), despite the use of MSICS, which has been shown to be a safe technique in LMIC [19-21].

These outcomes are comparable to those reported in other areas of the country. The RAAB study performed in Sofala Province reported good outcomes in 37.8% of the patients, which improved to 45.9% when considering PinVA [18]. The main causes of poor outcomes (VA <6/60) were selection errors (55.2%) and surgical complications (34.5%). The 2018 RAAB study conducted in the province of Nampula recorded 58.2% good outcomes and 22.4% poor outcomes in terms of PVA. The main causes of outcomes worse than 6/18 vision were a lack of optical correction (34%) and surgical complications (29%). The PinVA outcomes improved to 72.4% (good) and 16.3% (poor) [22].

The BOOST Cataract app does not collect data on moderate outcomes or their causes in phase 2. This is despite these outcomes representing a postoperative VA of <6/18 to ≥6/60, equating to moderate visual impairment (Table 1).

In this study, 33.8% (45/133) of the patients had a moderate outcome, and the main cause was refractive error. The magnitude of this finding seems to indicate that, in Inhambane Province, as in other LMIC, awareness of the causes of moderate outcomes is necessary to improve them [18,23,24]. Visual impairment due to residual refractive error may be reduced if stockpiles of IOLs in a range of powers and ocular biometry are accessible [25]. In our study, the mean postoperative spherical equivalent was higher than that in other studies [26,27], even though biometry was performed in 84.4% (119/141) of the eyes and 100% of the patients had undergone IOL implantation in the range of power accorded by biometry. Cooperation of patients with poor fixation of the operated eye may lead to errors when performing biometry.

The mean postoperative astigmatism was also higher than that reported in other studies [28]. In fact, 75.2% (85/113) of the eyes had astigmatism greater than 3D against the rule. The use of a superior scleral tunnel instead of a temporal tunnel may have produced astigmatism against the rule with a worse final VA outcome [29]. Additionally, the surgical material is often in poor condition due to excessive resterilization, which may lead to more superficial sclerocorneal incisions than desired, increasing induced surgical astigmatism risk [30].

### Strengths and Limitations

Although the sensitivity and specificity obtained allow reaching relevant conclusions on the real-world utility of the app at the study site, due to the small sample size, it is not possible to generalize the diagnostic accuracy of the BOOST Cataract app in other low-resource settings. In settings with a lower prevalence of suboptimal outcomes, the negative predictive value might be lower. Therefore, the app, if confirmed in larger studies, has to be restricted to settings with poor-resource health systems.

Data on surgical outcomes in Inhambane are scarce and only available from the RAAB study conducted 8 years ago. This was the first study to analyze the causes of poor and moderate outcomes in such depth.

Surgical quality criteria have recently been revised, and VA ≥6/12, representing mild visual impairment, has been adopted as the threshold for a good outcome [2]. Due to the introduction of the MSICS technique and widespread use of biometry, surgical outcomes have improved. This new threshold places higher demands on the expected outcomes and implies an improvement in the visual expectations of patients undergoing surgery in LMIC.

To analyze the diagnostic accuracy of the BOOST Cataract app, moderate and poor outcomes were grouped into the suboptimal outcome category. From a public health perspective, it is of interest to know the number of eyes that do not achieve a VA >6/60 after surgery, which is the level of severe visual impairment that has the greatest impact on patient quality of life because it affects their ability to walk independently. Grouping moderate and poor outcomes into suboptimal outcomes encourages improvements in both outcome categories without focusing only on poor outcomes.

### Conclusions

RAAB studies provide reliable data for planning eye health services and evaluating programs. These are the primary sources of information for measuring surgical outcomes at the population level in LMIC [31]. However, in settings with resource-poor eye health systems, the BOOST Cataract app offers the opportunity to assess outcomes at the local level; each center needs to analyze its surgical outcomes and develop specific strategies to improve quality.

## Abbreviations

BCVA: best corrected visual acuity
BOOST: Better Operative Software Outcomes Tool
IOL: intraocular lens
LMIC: low- and middle-income countries
MSICS: manual small-incision cataract surgery
PinVA: pinhole visual acuity
PVA: presenting visual acuity
RAAB: Rapid Assessment of Avoidable Blindness
VA: visual acuity
WHO: World Health Organization

## Data Availability

The data sets generated or analyzed during this study are available from the corresponding author on reasonable request. All relevant data are included in this article.
